# Messenger ribonucleic acid expression of KiSS-1 and serum level of kisspeptin in rat at different developmental stages

**DOI:** 10.1186/1687-9856-2015-S1-O28

**Published:** 2015-04-28

**Authors:** Jung Min Ahn, Ah Reum Kwon, Hyun Wook Chae, Kyungchul Song, Duk Hee Kim, Ho-Seong Kim

**Affiliations:** 1 Department of Pediatrics, Severance Children’s Hospital, Yonsei University College of Medicine, Seoul, Korea; 2 Sowha Children’s Hospital, Seoul, Korea

## Purpose

KiSS-1 and its product, kisspeptin is necessary for pubertal onset and proper adult gonadal function due to its stimulatory effect on the secretion of gonadotropin-releasing hormone (GnRH). Although the pathophysiological importance of KiSS-1 and kisspeptin is well known, the developmental patterns of expression of KiSS-1 genes and serum level of kisspeptin have not been explored to date. We report herein the expression profile of KiSS-1 genes and serum level of kisspeptin in the rat at different developmental stages.

## Methods

Spraque-Dawley (SD) strain female rats were used. To analyse expression of KiSS-1 mRNA, samples were obtained from hypothalamus, pituitary and ovaries in female rats according to developmental stages. At the same time, blood samples were collected for analysis of serum levels of kisspeptin and luteinizing hormone (LH). The expression of KiSS-1 mRNAs was assessed by RT-PCR and the serum levels of kisspeptin and LH were analyzed by ELISA.

## Results

The expressions of KiSS-1 gene in hypothalamus and ovary were increased according to developmental stages and peaked at the prepubertal stage (at day 27, respectively, 0.88 ± 0.22, 0.54 ± 0.25). However, there were no significant changes or correlations between developmental stages and KiSS-1 gene expression in pituitary. Serum kisspeptin level was also increased according to developmental stages. However, peak level of kisspeptin (35.43 ± 3.60 pg/mL) was in the pubertal stage at day 34. Serum LH level was also increased and peaked (23.29 ± 15.24 ng/mL) at pubertal stage (at day 38). However, an increasing pattern was little delayed than that of kisspeptin level.

## Conclusion

The expressions of KiSS-1 mRNA in hypothalamus and ovary, serum levels of kisspeptin and serum LH levels were increased according to developmental stages in rat in regular sequence. Therefore, serum kisspeptin levels can be an indication of KiSS-1 gene expression in hypothalamus and pubertal onset.

